# The Microbial Diversity of Non-Korean Kimchi as Revealed by Viable Counting and Metataxonomic Sequencing

**DOI:** 10.3390/foods9111568

**Published:** 2020-10-29

**Authors:** Antonietta Maoloni, Ilario Ferrocino, Vesna Milanović, Luca Cocolin, Maria Rita Corvaglia, Donatella Ottaviani, Chiara Bartolini, Giulia Talevi, Luca Belleggia, Federica Cardinali, Rico Marabini, Lucia Aquilanti, Andrea Osimani

**Affiliations:** 1 Dipartimento di Scienze Agrarie, Alimentari ed Ambientali, Università Politecnica delle Marche, via Brecce Bianche, 60131 Ancona, Italy; a.maoloni@pm.univpm.it (A.M.); v.milanovic@univpm.it (V.M.); l.belleggia@pm.univpm.it (L.B.); f.cardinali@staff.univpm.it (F.C.); anmicro@univpm.it (R.M.); 2 Department of Agricultural, Forest, and Food Science, University of Turin, Largo Paolo Braccini 2, Grugliasco, 10095 Torino, Italy; ilario.ferrocino@unito.it (I.F.); lucasimone.cocolin@unito.it (L.C.); mariarita.corvaglia@unito.it (M.R.C.); 3 Istituto Zooprofilattico Sperimentale dell’Umbria e delle Marche, Via Cupa di Posatora 3, 60131 Ancona, Italy; d.ottaviani@izsum.it (D.O.); c.bartolini@izsum.it (C.B.); g.talevi@izsum.it (G.T.)

**Keywords:** *Leuconostoc kimchii*, *Weissella soli*, *Candida sake*, fermented vegetables, microbial diversity

## Abstract

Kimchi is recognized worldwide as the flagship food of Korea. To date, most of the currently available microbiological studies on kimchi deal with Korean manufactures. Moreover, there is a lack of knowledge on the occurrence of eumycetes in kimchi. Given these premises, the present study was aimed at investigating the bacterial and fungal dynamics occurring during the natural fermentation of an artisan non-Korean kimchi manufacture. Lactic acid bacteria were dominant, while Enterobacteriaceae, Pseudomonadaceae, and yeasts progressively decreased during fermentation. *Erwinia* spp., *Pseudomonas* *veronii*, *Pseudomonas* *viridiflava*, *Rahnella* *aquatilis,* and *Sphingomonas* spp. were detected during the first 15 days of fermentation, whereas the last fermentation phase was dominated by *Leuconostoc kimchi*, together with *Weissella* *soli*. For the mycobiota at the beginning of the fermentation process, *Rhizoplaca* and *Pichia orientalis* were the dominant Operational Taxonomic Units (OTUs) in batch 1, whereas in batch 2 *Protomyces inundatus* prevailed. In the last stage of fermentation, *Saccharomyces cerevisiae*, *Candida sake,* *Penicillium*, and *Malassezia* were the most abundant taxa in both analyzed batches. The knowledge gained in the present study represents a step forward in the description of the microbial dynamics of kimchi produced outside the region of origin using local ingredients. It will also serve as a starting point for further isolation of kimchi-adapted microorganisms to be assayed as potential starters for the manufacturing of novel vegetable preserves with high quality and functional traits.

## 1. Introduction

Vegetable-based traditional fermented foods are manufactured worldwide but in some countries, they represent an important part of the daily diet. The most popular fermented vegetables include mudhika produced in Africa using cassava (*Manihot esculenta*) [1]; caxiri, tarubà, and yakupa produced in Latin America through cassava fermentation [2]; and sauerkraut, mainly produced in Central and Eastern European countries with shredded and salted cabbage [3]. Moreover, among the fermented vegetables that are recognized worldwide as unique kickshaws, paocai, jiangshui, miso, natto, tempeh, and kimchi represent further well-known masterpieces of Asian traditions [4].

Among vegetable-based fermented foods typically produced in Asia, kimchi is recognized as the flagship food of Korea [5]. Such a preparation, based on the fermentation of cabbage and/or other vegetables, was probably invented around 4000 years ago [6]. Moreover, during Confucianism, kimchi constituted the core of the ancestral worship table, together with rice and soup [4]. In 2013, the United Nations Educational, Scientific and Cultural Organization (UNESCO) recognized the cultural importance of kimchi and inscribed this Korean traditional preparation of vegetables in the Representative List of The Intangible Cultural Heritage of Humanity.

Kimchi is usually produced using cabbage, radish, and cucumber as main ingredients; moreover, different seasonings, including salts, garlic, leek, red pepper powder, and ginger are used in accordance with local traditions [7]. Actually, more than 200 varieties of kimchi are produced, each characterized by peculiar biochemical, nutritional, and sensory features, which are greatly affected by the raw ingredients (e.g., onion, Korean lettuce, sesame leaves, sweet potato vines, *Allium hookeri* Thwaites, deodeok, and water dropwort), the preparation method, and even region, seasonality, and cultural traditions [7,8]. Baechu (Chinese cabbage) kimchi is the most popular variety. Traditionally, the secret of making kimchi passed through generations from mothers to daughters [9]. However, as an effect of globalization, individuals and companies worldwide can now access the recipe for kimchi production. It is estimated that the worldwide market for kimchi will reach USD 3850 million in 2024, from USD 3000 million in 2019. Though in Italy kimchi is still far from being popular, various kimchi manufacturers and kimchi suppliers have been operating since the late 2010s.

Mature kimchi possesses unique and flavorful sensory traits, consisting of a combination of fresh, sour, spicy, hot, and sweet tastes [10]. It is generally low in calories and rich in vitamins (A, C, and B complex), various phytochemicals, minerals (calcium, iron, potassium), and dietary fiber [11].

From a microbiological point of view, mature kimchi is a closed ecosystem where complex microbial interactions occur. Kimchi fermentation is an anaerobic process during which the interactions between the microbiota and the raw materials lead to the production of microbial metabolites (e.g., mannitol, lactate, acetate, ethanol, exopolysaccharides, etc.) that strongly characterize the sensory traits of the product [12]. Of note, the production of organic acids from the carbohydrates’ metabolism results in a pH drop to 4.2–4.0 [13], which in turn contributes to the palatability and safety of this fermented vegetable-based food.

To the authors’ knowledge, most of the microbiological studies to date carried out on kimchi deal with the analysis of Korean manufactures, and no scientific data are currently available on non-Asian produced kimchi. A past taxonomic study carried out on Korean kimchi reported the prevalence of lactic acid bacteria as the main fermenting microorganisms, including *Leuconostoc*, *Lactobacillus*, *Lactococcus*, *Pediococcus,* and *Weissella* species [12]. More recently, a large-scale targeted metagenomic investigation focusing on the bacterial ecology of kimchi sampled during its natural fermentation showed the occurrence of a more complex and dynamic ecosystem, with the most adapted species prevailing at the end of the fermentation process [10].

Kimchi also represents an important source of candidate bacterial probiotics and protechnological strains that, due to their robustness, are very well adapted to the unfavorable conditions occurring in this food product [14]. A few lactic acid bacteria strains so far isolated from kimchi, such as *Lactobacillus acidophilus* KFRI342, *Lactococcus lactis* KC24, *L. plantarum* LPpnu, and *Leuconosctoc mesenteroides* LMpnu showed a potential anticancer activity [15,16,17], thus suggesting a possible beneficial effect of the consumption of kimchi on human health.

Unlike bacteria, there is a paucity of available data about the fungal dynamics occurring during kimchi fermentation. As recently reported by Kim et al. [18], in the early stages of fermentation low loads of yeasts can colonize the fermenting vegetable substrate, with a positive impact on the sensory traits of kimchi due to the production of fruity aromatic compounds and a mitigation of kimchi’s acidic and moldy flavors [18]. However, as reported by the same authors, high loads of yeasts (e.g., yeasts forming white colonies) occurring on kimchi*’s* surface, especially during the late phase of fermentation, can negatively affect the quality of the end product [18].

Given the above premises, the main aim of the present study was to explore the bacterial and fungal dynamics occurring during the natural fermentation of kimchi handcrafted by an artisan Italian manufacturer through conventional microbiological analyses (viable counting) and metataxonomic sequencing.

## 2. Materials and Methods

### 2.1. Kimchi Production

Two independent production batches of kimchi, referred to as batch 1 and batch 2, were prepared by a Korean staff at a small artisan producer located in Santa Maria Nuova (Ancona, Italy) using the following ingredients: Chinese cabbage 60.5 (g 100 g^−1^), turnip 13.5 (g 100 g^−1^), water 12.0 (g 100 g^−1^), onion 4.0 (g 100 g^−1^), pepper chili powder 2.0 (g 100 g^−1^), red pepper 2.0 (g 100 g^−1^), garlic leaves 2.0 (g 100 g^−1^), spring onion 1.0 (g 100 g^−1^), carrot 1.0 (g 100 g^−1^), ginger 0.5 (g 100 g^−1^), sucrose 1.0 (g 100 g^−1^), and salt 0.5 (g 100 g^−1^). All the vegetables were purchased from a local grocer.

**Figure 1:**
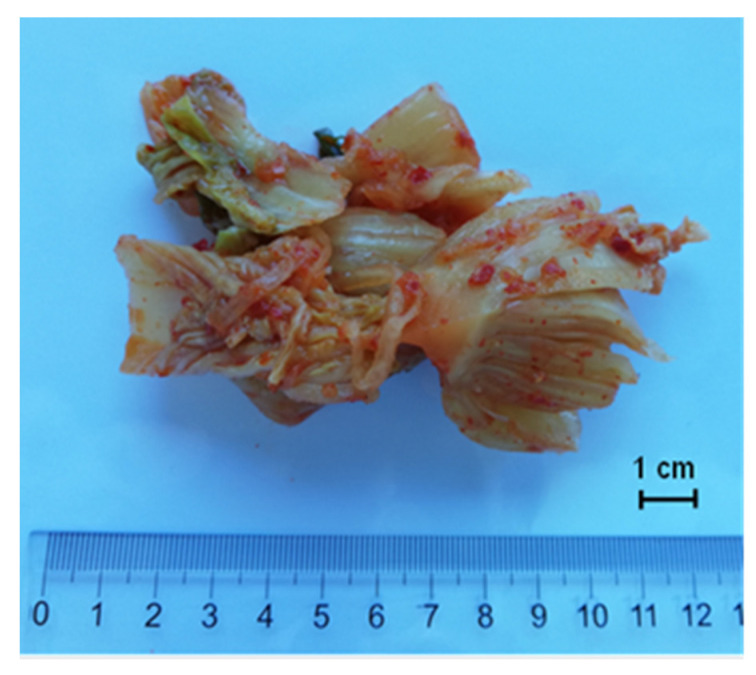
Ready-to-eat cabbage-origin kimchi.

Kimchi was produced according to the traditional Korean process described as follows. Briefly, the outer leaves were removed from the Chinese cabbage, trimmed, and then steeped in 10% (w v^−1^) NaCl for approximately 16–18 h at room temperature. The sauce used for cabbage dressing was obtained by mixing chopped vegetables (turnip, onion, carrot, ginger, spring onion, red peppers, and garlic leaves) with chili pepper powder, water, sugar, and salt. Thereafter, the sauce was stored under refrigerated conditions (5 °C) for approximately 16–18 h. The cabbage was rinsed, and the excess water drained. The sauce was carefully spread over the cabbage leaves. Kimchi was fermented at approximately 5 ± 1 °C for 57 days, up until a fixed pH of 4.2 was reached. Kimchi fermentation was carried out in a plastic box hermetically sealed with a lid. Samples of kimchi (Figure 1) were collected with sterile spoons immediately after preparation (t_0_) and after 2, 5, 15, 36, 43, 50, and 57 days of fermentation. The samples were transported to the laboratory under refrigerated conditions (4 °C) and processed immediately after arrival.

### 2.2. pH Determination

pH measurements were performed at the core of each kimchi sample as previously described by Belleggia et al. [19].

### 2.3. Microbial Counts

Ninety mL of sterile water containing 1 g L^−1^ bacteriological peptone were added to 10 g aliquots of each sample. The suspensions were homogenized for 2 min at 230 rpm in a Stomacher machine (400 Circulator, International PBI, Milan, Italy). Tenfold serial dilutions were prepared for the enumeration of: (i) mesophilic aerobic bacteria on Plate Count Agar incubated at 30 °C for 48 h; (ii) total mesophilic halophilic aerobic bacteria on Plate Count Agar added with 8% NaCl and incubated at 30 °C for 7 days; (iii) presumptive mesophilic lactobacilli on De Man, Rogosa, and Sharpe (MRS) agar incubated at 30 °C for 48 h; (iv) presumptive mesophilic lactococci on M17 agar incubated at 22 °C for 72 h; (v) halophilic lactobacilli on MRS agar added with 8% NaCl and incubated at 30 °C for 7 days; (vi) halophilic lactococci on M17 agar added with 8% NaCl and incubated at 22 °C for 10 days; (vii) Enterobacteriaceae on Violet Red Bile Glucose Agar (VRBGA) incubated at 37 °C for 24 h; (viii) Pseudomonadaceae enumerated on Pseudomonas Agar Base (PAB) supplemented with Cetrimide-Fucidin-Cephalosporin (CFC) selective supplement (VWR International, Milan, Italy) incubated at 30 °C for 24–48 h; (ix) yeasts counted on Rose Bengal Chloramphenicol Agar (RBCA) incubated at 25 °C for 72–96 h; (x) halophilic yeasts counted on RBCA agar added with 8% NaCl and incubated at 25 °C for 72 h. MRS agar was supplemented with cycloheximide (250 mg L^−1^) to inhibit the growth of eumycetes.

The results of viable counts were reported as mean value of two biological and three technical replicates expressed as the Log of colony-forming units (cfu) per gram of sample ± standard deviation.

Finally, the presence/absence of *Listeria monocytogenes* and *Salmonella* spp. was determined using a MINI VIDAS (Vitek Immunodiagnostic Assay System) apparatus (Biomerieux, Marcy l’Etoile, France) as previously described [19,20].

### 2.4. RNA Extraction and cDNA Synthesis

The 1.5 mL sample homogenates (10^−1^ dilution), prepared as described in Section 2.3, were centrifuged at 16,000 rpm for 10 min to obtain cell pellets subsequently covered with RNAlater Stabilization Solution (Ambion, Foster City, CA, USA) and stored at −80 °C until the extraction of RNA using E.Z.N.A. Bacterial RNA Kit (Omega Bio-tek, Norcross, GA, USA). The quantity, purity, and integrity of the extracted RNAs were checked as previously described by Garofalo et al. [21]. cDNA synthesis was performed using SensiFAST cDNA Synthesis Kit for RT-qPCR (Bioline, London, UK).

### 2.5. Metataxonomic Sequencing and Bioinformatics Analysis

cDNA was used for the amplification of bacterial 16S rRNA (V3/V4 region) [22] and fungal 26S rRNA genes (D1 domain) [23]. The sequencing of the purified PCR amplicons was performed in a MiSeq instrument in a 2 × 250 bp configuration, while QIIME v. 1.9 [24] was used for the analysis of the obtained reads. For bacteria, after Operational Taxonomic Units (OTUs) clustering at 99% of similarity, centroid sequences were mapped against the Greengenes 16S rRNA gene database, while for eumycetes the in-house database from Mota-Gutierrez et al. [23] was used. Taxonomic assignments were double-checked using BLAST suite tools. Chloroplast and mitochondria sequences were removed from the data sets. The OTUs table was rarefied at the lowest number of sequence/samples displaying the highest taxonomic resolution.

### 2.6. Data Analysis

The alpha diversity index was calculated by the VEGAN package of R. Diversity index, and OTUs table were used in R to find statistically significant differences in the samples as a function of the fermentation time. Principal Component Analysis (PCA), aimed at exploring relationships between experimental variables and detecting possible sample clusters, and one-way ANOVA analysis, used to analyze the effect of ripening time on the dependent variables for each batch separately, were performed as described by Belleggia et al. [19]

## 3. Results

### 3.1. pH Determination

**Figure 2:**
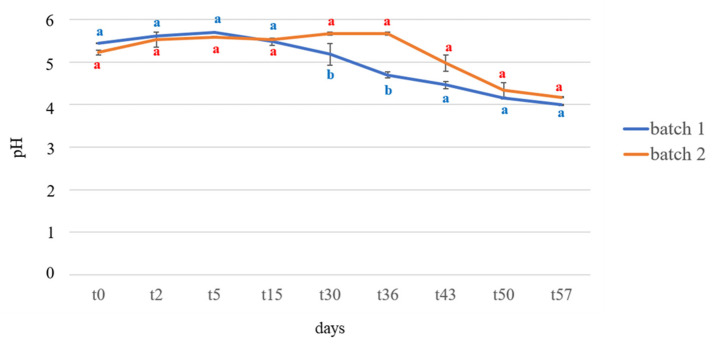
Results of pH measurements of two kimchi manufactures (batch 1 and batch 2) during fermentation. Values are expressed as means ± standard deviation. For each sampling time, means with different letters are significantly different (*p* < 0.05).

The results of pH measurements are shown in Figure 2. The values detected in the two analyzed batches had a similar trend, although kimchi produced in batch 1 was characterized by a faster pH reduction than batch 2. In more detail, at t_0_, the pH values were 5.44 ± 0.01 and 5.22 ± 0.06 for batch 1 and batch 2, respectively. A significant drop in pH was observed between t_30_ and t_36_ for batch 1, whereas a more progressive reduction was observed in batch 2, where a drop in pH was detected between t_36_ and t_43_. At t_57_ no significant differences were detected between the pH values of both batches, which tested at 3.99 ± 0.01 and 4.17 ± 0.01 for batch 1 and batch 2, respectively.

### 3.2. Microbiological Analyses

The results of viable counts performed on the two batches of kimchi are reported in Table 1. 

In more detail, the counts of mesophilic aerobic bacteria showed the same trend in both batches, with no significant differences between them.

For halophilic mesophilic aerobic bacteria, as well, at t_57_ no significant differences in both the analyzed batches were observed.

Regarding presumptive lactobacilli and presumptive halophilic lactobacilli, a progressive increase in the load of these microorganisms was seen during fermentation; the highest counts were observed from t_36_ to t_50_ in batch 1 and from t_43_ to t_57_ in batch 2.

Regarding presumptive lactococci and presumptive halophilic lactococci, the counts in samples of batches 1 and 2 showed no significant differences at t_57_.

Low Enterobacteriaceae counts were detected, with no significant differences between the two batches from t_43_ to t_57_.

As for Pseudomonadaceae, from t_36_ to t_57_, they were <1.0 Log cfu g^−1^ in both batches.

Regarding yeasts and halophilic yeasts, counts < 1.0 Log cfu g^−1^ were detected in both batches at t_57_.

Finally, *L. monocytogenes* or *Salmonella* spp. were never detected in 25 g of product, irrespective of the sampling time and the production batch.

### 3.3. Microbiota Diversity

**Figure 3:**
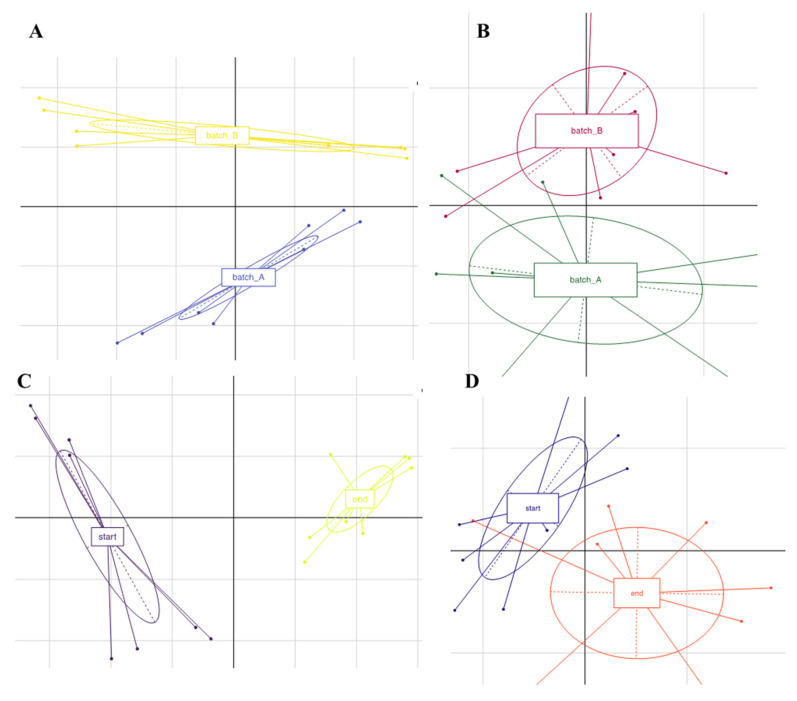
Principal Component Analysis (PCA) based on Operational Taxonomic Units (OTUs) relative abundance of kimchi samples for bacteria (panel **A**) and fungi (panel **B**) grouped according to the batch, or according to: (i) the fermentation period; (ii) beginning of fermentation: t0, t2, t5, t15; (iii) end of fermentation: t36, t43, t50, t57, for bacteria (panel **C**) and fungi (panel **D**).

**Figure 4:**
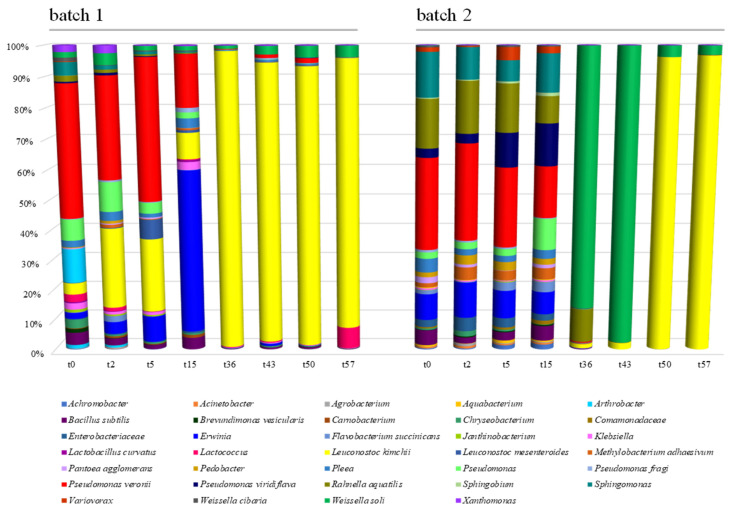
Relative abundance of bacterial Operational Taxonomic Units (OTUs) detected by sequencing in the analyzed kimchi batches. Samples are grouped according to batch (1 and 2) and labeled according to fermentation time.

**Figure 5:**
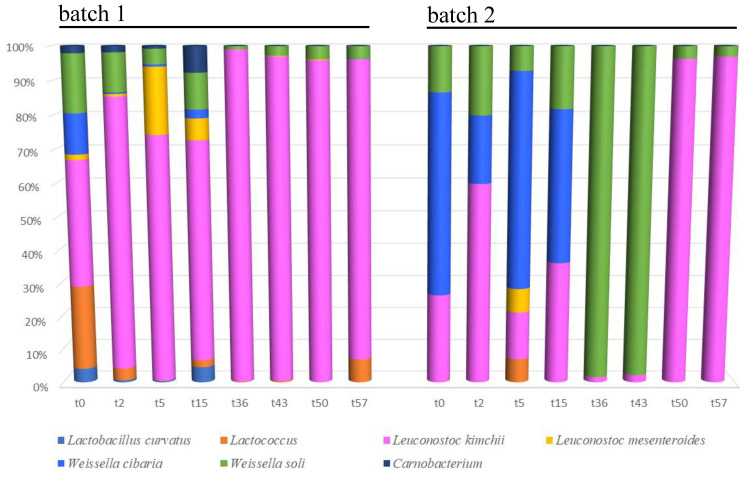
Relative abundance of lactic acid bacteria Operational Taxonomic Units (OTUs) detected by sequencing in the analyzed kimchi batches. Samples are grouped according to batch (1 and 2) and labeled according to fermentation time.

The total number of sequences used reach 94,353 reads, with an average value of 5585 reads/sample and a mean sequence length of 460 bp. Sample coverage calculation showed a satisfactory coverage for all the samples (>96%), whereas alpha diversity index did not show any significant difference as a function of the batch. Principal component analysis based on OTU table revealed a clear separation of the samples as a function of the batch (Figure 3, panel A). Samples analyzed at the beginning of the fermentation clustered together and were well separated from the samples at the end of this process (Figure 3, panel C). The microbiota composition at the highest taxonomic level (Figure 4) showed that, especially in batch 1, *Leuconostoc kimchii* was the dominant OTU after five days of fermentation (reaching almost 80% of the relative abundance up until the end of fermentation). Moreover, in batch 2, *Leuconostoc kimchii* was the dominant OTU only in the last two sampling points (reaching 90% of the relative abundance). Several spoilage microorganisms were identified in both the batches. In more detail, the presence of *Erwinia* spp. together with *Pseudomonas veronii*, *Pseudomonas viridiflava*, *Rahnella aquatilis,* and *Sphingomonas* spp. was observed during the first 15 days of fermentation. *Erwinia* spp. was predominant in batch 1 after 15 days of fermentation attesting at 28% of the relative abundance, whereas *P. veronii* was predominant in batch 1 in the first 15 days of fermentation (attesting at 25% and 16% of the relative abundance in batch 1 and 2, respectively). *P. viridiflava*, *Sphingomonas* spp., and *R. aquatilis* were predominant in batch 2 during the first 15 days of fermentation (with a relative abundance of 5%, 6% and 9%, respectively) (Figure 4). In addition, a high presence of *Weissella* soli was observed in batch 2 after 36 and 46 days of fermentation, reaching 90% of the relative abundance. Figure 5 shows the relative abundance of the sole lactic acid bacteria OTUs detected by sequencing in the two analyzed batches of kimchi.

**Figure 6:**
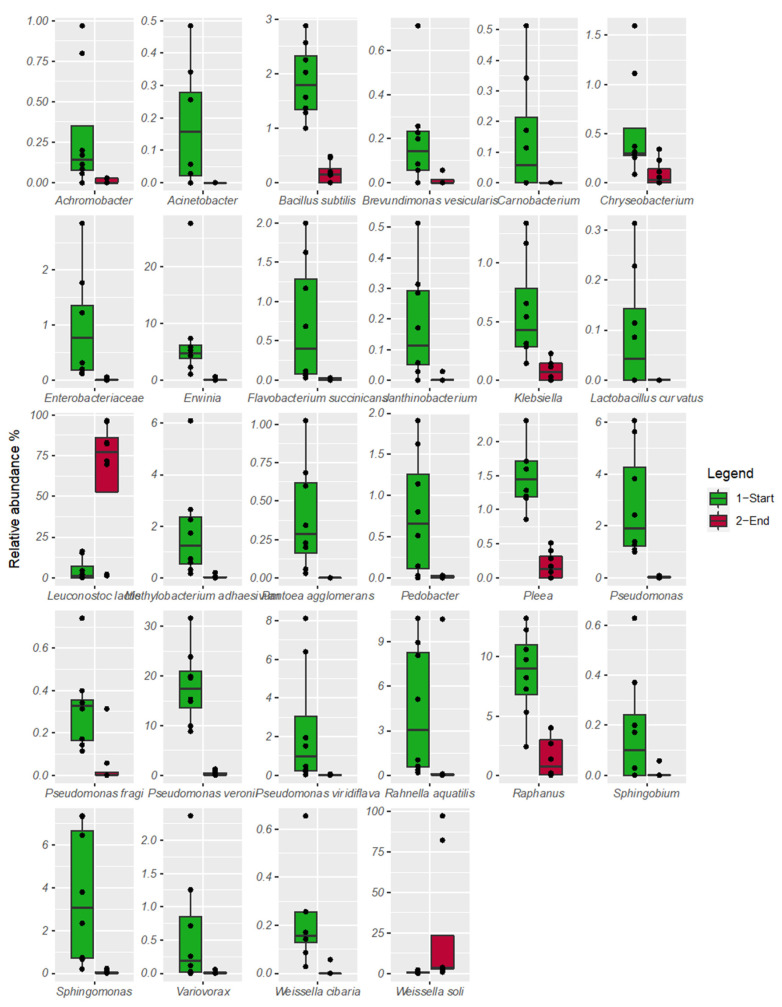
Boxplots showing the relative abundance of bacterial Operational Taxonomic Units (OTUs) between the beginning of fermentation: t0, t2, t5, t15, and the end of fermentation: t36, t43, t50, t57. Boxes represent the interquartile range (IQR) between the first and third quartiles, and the line inside represents the median (2nd quartile).

By comparing the relative abundance of the OTUs between the beginning and the end of fermentation, only *Weissella soli* and *Leuconostoc kimchii* appeared to be associated with the end of fermentation (FDR < 0.05), whereas the other OTUs (including the minor fraction with <1% of the relative species abundance) were associated with the early step of fermentation (Figure 6).

### 3.4. Mycobiota Diversity

The total number of sequences used for the downstream analysis reached 634,252 reads, with an average value of 40,910 reads/sample and a mean sequence length of 395 bp. Overall, a satisfactory sample coverage was showed by all the samples (>96%). Alpha diversity indexes were not significantly different according to time or batch.

PCA based on OTUs relative abundance of fungal taxa revealed two clusters separated according to batch and fermentation time (Figure 3, panel B and D, respectively).

**Figure 7:**
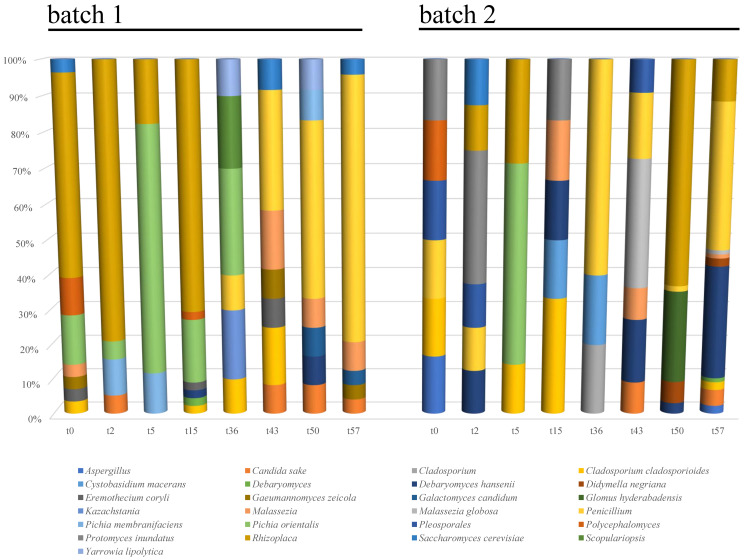
Relative abundance of fungal Operational Taxonomic Units (OTUs) detected by sequencing in the analyzed kimchi batches. Samples are grouped according to batch (1 and 2) and labeled according to fermentation time.

The mycobiota composition at the highest taxonomic level (Figure 7) showed an evolution across time, in both the analyzed batches, with significant differences between them. In batch 1, *Rhizoplaca* and *Pichia orientalis* were the dominant OTUs in the first 15 days of fermentation (with approximately 65% and 17% of the relative abundance, respectively), whereas, at the end of fermentation, *Penicillium*, *Candida sake, Malassezia*, and *Saccharomyces cerevisiae* were the most abundant taxa, reaching 67, 8, 7, and 3% of the relative species abundance, respectively.

In batch 2, a stable occurrence of *Penicillium* spp. was detected, especially at the end of the fermentation (when it reached 40% of the relative abundance), together with *Rhizoplaca* spp. (58% and 11% of the relative abundance, after 50 and 57 days, respectively) (Figure 7). *Protomyces inundatus* was predominant after 2 days of fermentation, with 30% relative abundance, whereas *Glomus hyderabadensis* and *Debaromyces hansenii* were predominant after 50 and 57 days of fermentation, reaching 24% and 31% of the relative abundance, respectively. *Malassezia* and *Candida sake* were also detected as minority taxa (<10% of the relative abundance) at the end of fermentation.

**Figure 8:**
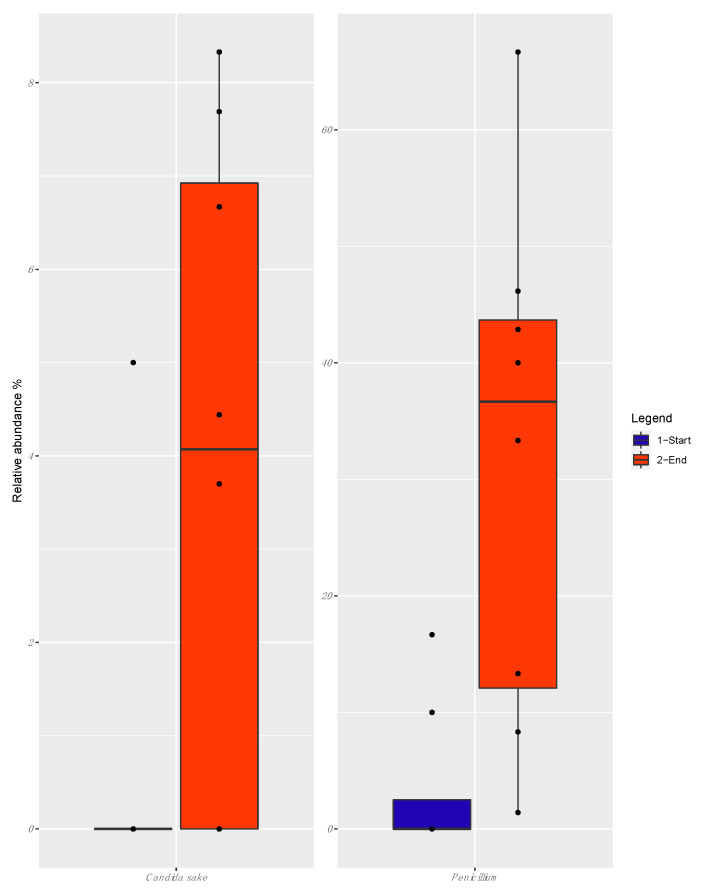
Boxplots showing the relative abundance of fungal Operational Taxonomic Units (OTUs) between the beginning of fermentation: t0, t2, t5, t15, and the end of fermentation: t36, t43, t50, t57. Boxes represent the interquartile range (IQR) between the first and third quartiles, and the line inside represents the median (2nd quartile).

When the relative abundance of the OTUs at the beginning and the end of the fermentation were compared, only *Penicillium* and *Candida sake* appeared to be associated with the end of fermentation (FDR < 0.05) (Figure 8).

## 4. Discussion

As reviewed by Chang [25], kimchi is produced through a spontaneous fermentation, generally lasting from a few weeks (e.g., four) to months (e.g., three); this fermentation process is mainly mediated by lactic acid bacteria naturally occurring in the raw materials and the production environment.

Regarding the samples of kimchi analyzed in the present study, pH values detected in both batches at t_0_ were in accordance with those reported for Korean kimchi at the beginning of fermentation, attesting between 5.0 and 5.4, depending on the raw material used [10,12]. However, a different evolution of this parameter was seen during the fermentation with respect to Korean kimchi, which reaches a pH of ~4.0 as early as 4 weeks after fermentation [10,26]. Indeed, in the samples herein analyzed, a pH value of about 4.0 was reached after 57 days of fermentation. It is noteworthy that the type of raw material and its natural contamination can strongly affect the evolution of pH during kimchi fermentation; in this regard, Song et al. [27] have recently reported different pH values for mature cabbage-, garlic-, ginger-, and red pepper-origin kimchi. Interestingly, the pH values detected in the kimchi manufacture under study were in accordance with those reported for cabbage-kimchi.

As a general trend, viable counts suggested the establishment of an active microbial community composed mainly of mesophilic aerobes and lactic acid bacteria; in contrast, progressively decreasing counts of Enterobacteriaceae, Pseudomonadaceae, and yeasts were seen during kimchi fermentation.

Regarding viable counts of total mesophilic aerobes, enumerated on growth media with or without the addition of 3% (w/v) NaCl, similar trends were observed between the two analyzed batches. In more detail, almost stable loads attesting at 5 Log cfu g^−1^ were seen during the whole fermentation period. The values detected at t_0_ were in accordance with those reported by Lee et al. [10] in the raw materials used for kimchi manufactured in Gyeong-gi province of Korea. By contrast, the counts of total mesophilic aerobes were lower than those reported in Korean kimchi by Hong et al. [28], attesting at >8 Log cfu g^−1^ after 9 days of fermentation. Interestingly, Hong et al. [28] reported a linear association between the fermentation temperature and loads of total mesophilic aerobes, with a slowdown of the microbial growth at 4 °C.

Regarding lactic acid bacteria counts, two different trends were seen in the two production batches, feasibly due to the differences occurring in the microbiota composition of the vegetables used as ingredients or the microbial interactions established during fermentation. In more detail, in batch 1 the load of lactobacilli progressively increased up until day 36, when it reached a maximum mean value of 6 Log cfu g^−1^. In batch 2, the load of lactobacilli remained almost stable during the early stage of fermentation (first 15 days), which was followed by a rapid increase. Markedly higher viable cell counts of lactobacilli, attesting at about 9 Log cfu g^−1^ were reported by Hong et al. [28] in a previous research dealing with the microbial dynamics of kimchi during its natural fermentation. Lactic acid bacteria counts detected in the present study were also lower that those reported by Lee et al. [10] in 88 kimchi manufactures sampled from six provinces (Chungcheong, Gangwon, Gyeonggi, Gyeongsang, Jeolla, and Jeju) in South Korea.

In accordance with lactic acid bacteria increase, in both production batches a progressive decrease of Enterobacteriaceae counts was seen, with a complete disappearance of the latter microorganisms at days 36 and 50 in batches 1 and 2, respectively. As previously found in numerous fermented vegetables, the acidity due to accumulation of organic acids produced by lactic acid bacteria was mainly responsible for the inhibition and death of Enterobacteriaceae [29,30,31,32]. Members of this family are acknowledged as indicators of process hygiene, hence, their load in foods should be carefully assessed [33].

Relatively high counts of Pseudomonadaceae were detected at early stages of fermentation (from day 0 to 15). This finding agrees well with the results reported by Park et al. [34] about the possible dominance of pseudomonads during the first stages of kimchi fermentation. The counts of Pseudomonadaceae were also in accordance with those reported by Wouters et al. [35] in raw materials used for the production of spontaneously fermented leek, attesting at about 5 Log cfu g^−1^ and progressively disappearing during the first week of fermentation.

Finally, viable counts of yeasts on Rose Bengal Agar without or with 3% (w/v) NaCl showed a similar decreasing trend in both production batches, with values below the limit of detection at the late stage of fermentation. Data herein obtained are in accordance with the occurrence of yeasts in kimchi already reported by Kim et al. [18], whereas a quite different picture emerged from the study of Jeong et al. [26], who reported an increase in the load of *Saccharomyces* spp. during fermentation of kimchi, from day 19 up until day 45. Moreover, Chang et al. [36] reported the isolation of yeasts such as *Pichia* spp. from mature kimchi. Since yeasts are halotolerant and acidophilic microorganisms that are frequently isolated from acidic fermented vegetables and brine [14], further research is needed to better elucidate the scarce occurrence of yeasts in the kimchi manufacture herein analyzed.

The results of metataxonomic analyses highlighted the presence of vegetable-associated bacteria with a progressive reduction of spoilage taxa counterbalanced by a progressive increase of pro-technological taxa mainly ascribed to the lactic acid bacteria group, which dominated from t_36_ to t_57_.

Regarding OTUs that initially characterized the analyzed kimchi, *Pseudomonas* species were detected in samples collected from both batches from t_0_ to t_15_. *Pseudomonas* species had already been detected by Lee et al. [8] in samples of Korean kimchi; moreover, as reported by Song et al. [27], pseudomonads were among the dominating taxa during the early fermentation of cabbage-origin Korean kimchi. Interestingly, in the present study, the decrease of the relative abundance of *Pseudomonas* spp. during fermentation was in agreement with the progressive decrease of viable counts of pseudomonads, thus confirming the profitability of combining culture-dependent with culture-independent methods. Regarding the occurrence of *P. veronii*, this species was first isolated from natural mineral waters [37] and more recently found as part of the core microbiota of fresh-cut produce processing facilities, together with *P. viridiflava* [38]. Moreover, both *P. veronii* and *P. viridiflava* were reported to be the causative agents of soft rot in carrot [39], thus suggesting carrots as a potential source of contamination by these two species in the analyzed kimchi.

During the early stage of kimchi fermentation (from t_0_ to t_15_), *R. aquatilis* was abundantly detected in samples from batch 2. This epiphytic bacterium can be frequently isolated from water, plant leaves and fruit, soil, foods as well as non-environmental samples such as blood, bronchial washings, wounds, and urine [40]. *Rahnella* species have already been detected in the microbiota of 25 cabbage-origin kimchi samples from traditional Korean temples that produce traditional temple style food [41]. These microorganisms were also detected in a large-scale metagenomics study carried out on 88 Korean kimchi sampled during fermentation [10], thus confirming the adaptation of this bacterial genus to the kimchi environment.

Regarding the presence of *Erwinia* spp., this bacterial genus, which is included in the Enterobacteriaceae family, was detected until t_15_. *Erwinia* encompasses species with cellulolytic and pectolytic activities that produce the soft rot of potato tubers [42]. The occurrence of *Erwinia* spp. in the early stage of fermentation of Korean white kimchi was already reported by Park et al. [43], whereas the same genus was also found by Park et al. [34] in 10 industrial-scale batches of Korean kimchi.

As for the presence of *Sphingomonas* spp., more than 100 species belonging to this bacterial genus have already been isolated from various environmental sources, including phyllosphere, rhizosphere, and plant roots [44]. Moreover, this genus was detected by Jung et al. [45] during the early stage of Korean kimchi fermentation [45].

Between t_15_ and t_36_, a clear shift in the relative abundance of the microbial species emerged, with an almost complete replacement of spoilage species with lactic acid bacteria, mainly represented by *W. soli* and *L. kimchi*. This finding suggests the establishment during fermentation of unfavorable environmental conditions for the survival of undesired bacteria.

Regarding *W. soli*, principally detected in samples from batch 2, this lactic acid bacteria species belonging to the Leuconostoccaceae family was first isolated from the soil environment by Magnusson et al. [46]. *W. soli* possesses a heterofermentative metabolism that, from glucose fermentation, produces lactic acid, CO_2_, ethanol, and/or acetate. Moreover, some *Weissella* strains, including *Weissella cibaria*, also detected in this study, were found to produce bacteriocins, such as weissellin, weissellicin 110, and weissellicin D, L., M and Y [47]. Although several *Weissella* species were abundantly detected in Korean kimchi [10], to the authors’ knowledge *W. soli* has rarely been isolated from this food product, where it was found as a minor species by Jeong et al. [26]. Although there is a lack of knowledge on the role of *W. soli* in kimchi, it is noteworthy that *Weissella* strains isolated from this fermented vegetable have recently shown potential prophylactic properties and probiotic features, being able to tolerate artificial gastric juice and bile salts and showing a high binding capacity for intestinal epithelial cells [48].

*Leuconostoc kimchii* was found to dominate in both analyzed batches at the end of fermentation (from t_50_ to t_57_). This microorganism was first isolated from kimchi manufactured in Korea, thus suggesting its strong adaptation to this fermented vegetable [49,50,51]. The use of *L. kimchi* strains as starter cultures for kimchi production was previously evaluated thanks to the potential health benefits for the consumers (e.g., lipid metabolism regulation) [51]. Interestingly, a recent study carried out on diet-induced obese mice reported that in animals administered with *L. kimchii* GJ2 isolated from Korean kimchi a significant decrease in hepatic triglyceride and fatty acid content was observed [52]. Moreover, Jo et al. [53] reported that kimchi fermented with the strain *L. kimchii* GJ2 provided efficient cholesterol-lowering effects in rats fed with a high-fat and high-cholesterol diet, thus suggesting the suitable use of this strain for the production of kimchi with functional features. Consistent with the well-known exopolysaccharides (EPS)-producing capabilities of leuconostocs, the production of dextran and levan with potential prebiotic activity by *L. kimchi* was reported by Schleifer [54] and Torres-Rodríguez et al. [55]. More recently, Rizzello et al. [56] have characterized the EPS production ability and other technological features of a *L. kimchi* strain for its exploitation as a starter culture for legumes fermentation, thus suggesting the potential suitability of this species for the manufacturing of novel vegetable-based fermented foods.

The metataxonomic analyses applied directly to the food matrix have allowed major and minor fungal taxa to be also detected, thus representing a further step toward the knowledge of the mycobiota of kimchi. As a general trend, *Rhizoplaca* spp. and *P. orientalis* were found to dominate the early stage of kimchi fermentation.

*Rhizoplaca* is a genus of foliose lichenized fungi (e.g., *Rhizoplaca melanophthalma*) in the family Lecanoraceae, including several morphologically distinct species that are geographically and ecologically widespread [57].

As for the presence of *P. orientalis* (Syn. *Issatchenkia orientalis*), yeasts in the genus *Pichia* were already isolated from mature kimchi characterized by low pH values [36]. In this regard, *Pichia kluyveri* was detected by Chang et al. [36] in Korean kimchi, whereas the osmotolerant yeast *Pichia guilliermondii* was detected in waste brine generated from kimchi production [58]. As reported by Chang et al. [36], the growth of *P. orientalis* is generally suppressed during kimchi fermentation, thus explaining its dominance in the sole early stage fermentation in both batches herein analyzed.

In the present study, *Penicillium* spp., *S. cerevisiae*, and *C. sake* were associated with the late stage of fermentation. However, the low load of eumycetes (<1 Log cfu g^−1^) found in both analyzed batches at the end of fermentation suggests the occurrence of the detected taxa at very low levels or in the state of viable but not culturable cells.

Interestingly, the occurrence of both *Saccharomyces* and *Candida* species has already been reported by Jeong et al. [26] during the late stage of fermentation of dongchimi, the traditional Korean watery kimchi. As suggested by Chang et al. [36], these yeasts can grow in kimchi at pH values between 4.0 and 7.0; moreover, white colony-forming yeasts, such as *C. sake* and *Debaryomyces* spp., were also detected in packed kimchi stored in refrigerated conditions [18], thus confirming their adaptation to cold environments.

## 5. Conclusions

Kimchi is one of the food products that best represent the tradition of the Korean cuisine and possess functional and health-promoting features related mainly to the metabolic activity of lactic acid bacteria. Based on the overall results herein collected, the non-Korean kimchi manufacture analyzed was characterized by microbial populations and dynamics that greatly overlapped those of more well-known Korean “relatives.” Indeed, typical kimchi-associated lactic acid bacteria species were surprisingly detected as dominant taxa in both batches analyzed, thus confirming their high adaptation to kimchi raw materials and production process. As already revealed by the available scientific literature, lactic acid bacteria isolated from kimchi can likely be used as starter cultures to produce novel functional fermented vegetables. As a matter of fact, lactic acid bacteria occurring in kimchi are well adapted to salty environments, and this feature can be extremely advantageous for their exploitation as starters for the fermentation of vegetables in brine. Given this premise, the knowledge gained in the present study represents a step forward in the description of the microbial dynamics of kimchi produced outside the region of origin using local ingredients. At this regard, the results collected compared to those available in the scientific literature for Korean kimchi manufactures seem to suggest a neatly higher effect of process parameters rather than the adventitious microbial populations on the shaping of mature kimchi microbiota. The evidences emerged from the present study will also serve as a starting point for further isolation of vegetable-adapted lactic acid bacteria to be assayed as potential starters for the manufacturing of novel vegetable preserves with high quality and functional traits. The present study also highlighted the presence of fungal taxa, whose contribution to the sensory traits of kimchi must be further investigated.

## Figures and Tables

**Table 1:** Results of viable counting (Log cfu g^−1^) of bacteria and eumycetes in kimchi during fermentation.

Batch	Sampling Time (Days)	Mesophilic Aerobic Bacteria	Halophilic Mesophilic Aerobic Bacteria	Presumptive Lactobacilli	Presumptive Halophilic Lactobacilli	Presumptive Lactococci	Presumptive Halophilic Lactococci	Enterobacteriaceae	Yeasts	Halophilic Yeasts	Pseudomonadaceae
1	t0	5.2 ± 0.1 ^a^	5.3 ± 0.1 ^a^	3.5 ± 0.1 ^b^	3.5 ± 0.1 ^b^	5.0 ± 0.1 ^a^	4.9 ± 0.0 ^a^	1.7 ± 0.3 ^b^	1.3 ± 0.0 ^a^	<1.0 ^b^	4.5 ± 0.2 ^a^
	t2	5.3 ± 0.0 ^a^	5.3 ± 0.1 ^a^	3.6 ± 0.5 ^b^	3.5 ± 0.5 ^b^	5.0 ± 0.1 ^a^	5.1 ± 0.1 ^a^	2.3 ± 0.6 ^b^	1.4 ± 0.1 ^a^	1.4 ± 0.1 ^a^	5.2 ± 0.1 ^a^
	t5	5.2 ± 0.0 ^a^	5.2 ± 0.0 ^a^	4.2 ± 0.0 ^b^	4.2 ± 0.0 ^b^	5.0 ± 0.1 ^a^	5.1 ± 0.2 ^a^	3.5 ± 0.5 ^a^	1.1 ± 0.1 ^a^	1.2 ± 0.3 ^a^	4.8 ± 0.3 ^a^
	t15	5.3 ± 0.1 ^a^	5.3 ± 0.0 ^a^	4.8 ± 0.2 ^b^	4.8 ± 0.1 ^b^	5.2 ± 0.0 ^a^	4.8 ± 0.1 ^a^	3.4 ± 0.2 ^a^	<1.0 ^b^	<1.0 ^b^	5.1 ± 0.1 ^a^
	t36	6.3 ± 0.1 ^a^	5.9 ± 0.2 ^a^	6.3 ± 0.0 ^a^	6.2 ± 0.2 ^a^	5.0 ± 0.2 ^a^	4.9 ± 0.2 ^a^	<1.0 ^c^	<1.0 ^b^	<1.0 ^b^	<1.0 ^b^
	t43	5.6 ± 0.0 ^a^	5.3 ± 0.0 ^a^	6.3 ± 0.1 ^a^	6.4 ± 0.1 ^a^	5.0 ± 0.2 ^a^	5.1 ± 0.1 ^a^	<1.0 ^c^	<1.0 ^b^	<1.0 ^b^	<1.0 ^b^
	t50	5.5 ± 0.0 ^a^	5.4 ± 0.0 ^a^	6.2 ± 0.2 a	5.6 ± 0.1 ^a^	4.9 ± 0.3 ^a^	5.0 ± 0.1 ^a^	<1.0 ^c^	<1.0 ^b^	<1.0 ^b^	<1.0 ^b^
	t57	5.4 ± 0.0 ^a^	5.3 ± 0.0 ^a^	4.6 ± 0.1 ^b^	4.7 ± 0.1 ^b^	5.0 ± 0.2 ^a^	5.0 ± 0.1 ^a^	<1.0 ^c^	<1.0 ^b^	<1.0 ^b^	<1.0 ^b^
2	t0	5.7 ± 0.3 ^a^	5.6 ± 0.2 ^a^	2.1 ± 0.2 ^c^	2.4 ± 0.0 ^b^	5.5 ± 0.3 ^a^	5.4 ± 0.1 ^a^	2.8 ± 0.2 ^a^	2.5 ± 0.1 ^a^	1.9 ± 0.0 ^a^	4.9 ± 0.1 ^a^
	t2	5.3 ± 0.1 ^a^	5.3 ± 0.0 ^a^	1.5 ± 0.1 ^c^	1.4 ± 0.2 ^b^	5.2 ± 0.1 ^a^	5.1 ± 0.2 ^a^	2.0 ± 0.2 ^a^	2.1 ± 0.0 ^a^	1.7 ± 0.0 ^a^	4.2 ± 0.0 ^a^
	t5	5.2 ± 0.1 ^a^	5.2 ± 0.1 ^a^	1.2 ± 0.0 ^c^	1.2 ± 0.1 ^b^	5.0 ± 0.1 ^a^	4.8 ± 0.1 ^a^	1.8 ± 0.1 ^a^	1.5 ± 0.2 ^b^	1.0 ± 0.2 ^b^	4.9 ± 0.0 ^a^
	t15	5.1 ± 0.1 ^a^	4.8 ± 0.1 ^a^	2.0 ± 0.1 ^c^	1.5 ± 0.1 ^b^	4.9 ± 0.0 ^a^	4.4 ± 0.0 ^a^	2.4 ± 0.3 ^a^	1.0 ± 0.0 ^b^	< 1.0 ^b^	4.9 ± 0.0 ^a^
	t36	5.1 ± 0.1 ^a^	5.1 ± 0.2 ^a^	4.8 ± 0.7 ^b^	2.5 ± 0.8 ^b^	4.8 ± 0.0 ^a^	4.3 ± 0.4 ^a^	1.6 ± 0.6 ^a^	<1.0 ^c^	<1.0 ^b^	<1.0 ^b^
	t43	4.9 ± 0.0 ^a^	4.8 ± 0.2 ^a^	7.3 ± 0.2 ^a^	4.2 ± 0.3 ^a^	4.5 ± 0.3 ^a^	4.4 ± 0.3 ^a^	<1.0 ^c^	<1.0 ^c^	<1.0 ^b^	<1.0 ^b^
	t50	4.9 ± 0.0 ^a^	4.7 ± 0.0 ^a^	7.3 ± 0.0 ^a^	5.0 ± 0.4 ^a^	4.6 ± 0.1 ^a^	4.6 ± 0.0 ^a^	<1.0 ^c^	<1.0 ^c^	<1.0 ^b^	<1.0 ^b^
	t57	4.9 ± 0.1 ^a^	4.9 ± 0.1 ^a^	6.4 ± 0.5 ^a^	4.3 ± 0.1 ^a^	4.2 ± 0.1 ^a^	4.2 ± 0.1 ^a^	<1.0 ^c^	<1.0 ^c^	<1.0 ^b^	<1.0 ^b^

Note: cfu, colony-forming units; Values are expressed as means ± standard deviation; For each batch, within each column, means with different superscript letters are significantly different (*p* < 0.05).
